# Estimating gene regulatory networks with pandaR

**DOI:** 10.1093/bioinformatics/btx139

**Published:** 2017-03-11

**Authors:** Daniel Schlauch, Joseph N Paulson, Albert Young, Kimberly Glass, John Quackenbush

**Affiliations:** 1 Department of Computational Biology and Biostatistics, Dana-Farber Cancer Institute, Brigham and Women’s Hospital, Boston, MA, USA; 2 Department of Biostatistics, Harvard T.H. Chan School of Public Health, Brigham and Women’s Hospital, Boston, MA, USA; 3 Channing Division of Network Medicine, Brigham and Women’s Hospital, Boston, MA, USA

## Abstract

PANDA (Passing Attributes between Networks for Data Assimilation) is a gene regulatory network inference method that begins with a model of transcription factor–target gene interactions and uses message passing to update the network model given available transcriptomic and protein–protein interaction data. PANDA is used to estimate networks for each experimental group and the network models are then compared between groups to explore transcriptional processes that distinguish the groups. We present pandaR (bioconductor.org/packages/pandaR), a Bioconductor package that implements PANDA and provides a framework for exploratory data analysis on gene regulatory networks.

**Availability and Implementation:** PandaR is provided as a Bioconductor R Package and is available at bioconductor.org/packages/pandaR.

## 1 Introduction

While correlation-based networks are widely used in transcriptomic data analysis, such networks do not explicitly model the biological mechanisms involved in regulating gene expression, such as the binding of transcription factors (TFs) to the regulatory regions of a gene. Passing Attributes between Networks for Data Assimilation (PANDA) (Glass *et al.*, 2013) is an integrative network inference method that explicitly models interactions between TFs and their putative target genes. PANDA starts with an initial network model derived from motif-based TF–target mapping to the genome, and uses a message-passing framework to refine that initial model in each phenotype given gene expression and other data. PANDA does not directly incorporate co-expression information between regulators and targets. Instead, edges in PANDA networks reflect the overall consistency between a TF’s regulatory profile with the target gene’s co-expression. In a number of applications, PANDA has provided insight into the regulatory context of genes and TFs associated with disease and other phenotypes (Glass *et al.* 2014, 2015; Lao *et al.*, 2015; Vargas *et al.*, 2016).

## 2 Materials and methods

### 2.1 PANDA

**Fig. 1:**
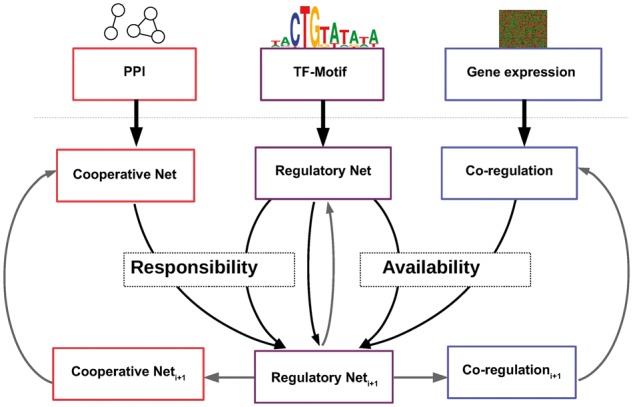
The PANDA algorithm takes as input protein–protein interaction data, a transcription factor–gene interaction network prior and gene expression data. Three networks representing inferred TF–TF co-operativity, TF–gene regulatory processes and gene co-regulation are then iteratively updated using message passing until the model converges

PANDA’s regulatory network model is fundamentally a bipartite graph in which TFs are connected to target genes. In PANDA’s message passing model, the edge weights are calculated based on the evidence that information from a particular TF is successfully passed to a particular gene. This evidence comes from the agreement between two estimated quantities on each edge, referred to as the *availability* and the *responsibility*(Fig. 1).

The *availability* is an estimate of the responsiveness of a gene *j* to TF *i*. The assumption in calculating the *availability* is that genes with correlated expression are likely to be regulated by a common TF. Hence, the *availability* is based on correlation in expression between gene *j* and other genes with the strength of evidence for regulatory interactions (edges) between TF *i* and other genes. Analogous to this, the *responsibility* is an estimate of the influence of TF *i* on gene *j*, and models the fact that TFs that form a complex are more likely to regulate the same target gene. The estimated *responsibility* is therefore based on the concordance between the set of TFs known to interact with TF *i* (based on protein–protein interaction (PPI) data) and the respective strength of evidence of a regulatory association between those other TFs and gene *j*. Both the *availability* and *responsibility* are estimated using a modified version of the Tanimoto similarity. We define this similarity between two nodes, *i* and *j*, as $T_{i,j}=\frac{\sum_{k}x_{k}y_{k}}{\sqrt{\sum_{k}x_{k}^{2}+\sum_{k}y_{k}^{2}-|\sum_{k}x_{k}y_{k}|}}$. In the case of *availability*, *x_k_* is the correlation between gene *j* and gene *k*, and *y_k_* is the edge weight between TF *i* and gene *k*. In the case of *responsibility x_k_* represents whether a PPI exists between TF *i* and TF *k*, and *y_k_* is the edge weight between TF *k* and gene *j*.

The edge weight between TF *i* and gene *j* is then updated based on the mean of the *responsibility* and *availability*. PANDA iteratively estimates edge weights until the process converges to the most parsimonious structure for information flow given the data. The result is a network model, represented by edge weights for every pairwise combination of TFs and genes, based on evidence from gene expression, sequence motif and PPI data. In analysis of simulated data, and data from systems in which confirmatory ChIP evidence are available, PANDA has been shown to outperform other competing methods (Glass *et al.*, 2013).

pandaR implements the PANDA algorithm in an easy-to-use Bioconductor package. Beginning with gene expression, TF gene interaction priors, and optional PPIs, pandaR generates a regulatory network for *m* TFs regulating *n* genes and presents it as an *m *×* n* adjacency matrix. It also provides the user with an estimated TF-by-TF ‘cooperativity network’ and gene-by-gene ‘co-regulation network’, both estimated by PANDA and reported as complete graphs representing the evidence for TF cooperation and gene co-regulation, respectively.

pandaR also provides a number of exploratory data visualizations of the inferred network’s properties and diagnostic tools. Our hypothesis in developing PANDA was that gene regulatory networks differ between biological states and that changes in the network are linked to phenotypic differences. Therefore, the pandaR package extends the PANDA network inference model by including a number of functions that can aide in comparing network structures between phenotypes. For example, the function plotGraph(topSubnet) integrates with igraph to generate a bipartite visualization of the PANDA networks. Because networks are often organized into functionally coherent communities, users can investigate and plot community structure using plotCommunityDetection(topNet). Additional network comparison functions include plotZ(pandaResultControl, pandaResultCase), which presents a scatterplot of the edge weights between two inferred networks. This function integrates with ggplot2, allowing the user to define graphics based on genes and TFs to easily identify functionally relevant sets of differential edge weights. We also implemented a function calcDegreeDifference() to calculate a gene’s degree or the degree difference between regulatory networks. Since it is important to benchmark the predicted edges of pandaR and compare its performance with alternative methods, we have included a function, validateNetwork(), which integrates the package ROCR and can be used to compare PANDA’s network inference results against a known reference standard. Finally, users can also use the function lioness(), which uses a unique linear interpolation method to estimate network models for each individual sample in a population (Kuijjer *et al.*, 2015). Unlike other methods that project gene expression onto an existing network, the lioness() function uses a leave-one-out method to estimate each individual edge weight in the network.

### 2.2 Data input

pandaR accepts input data in a variety of formats. Gene expression data can be input as either a data.frame, matrix, or as a Bioconductor ExpressionSet. TF gene interaction priors for the regulatory network can be input as a matrix or data.frame, with triplet columns specifying a putative regulatory edge from a TF (column 1) to gene (column 2) with a defined weight (column 3), typically initialized as 1.0; the regulatory prior is generally based on mapping TF motifs to target genes based on genomic sequence information. PPI data are not required but can be input as a either a matrix or data.frame that includes protein pairs and an interaction weight. Annotation type is not restricted except that node names for genes in the regulatory file must match node names in the gene expression file and TF names must match in both the regulatory and PPI inputs.

### 2.3 Example

An example data set generated from a subset of human gene expression data is available by running: data(pandaToyData).

The primary function in pandaR is called using

pandaResult <– panda(pandaToyData$motif,

pandaToyData$expression, pandaToyData$ppi)

where pandaResult is a ‘panda’ object that contains matrices describing the complete bipartite gene regulatory network and complete networks for gene co-regulation and TF cooperation. Due to the completeness of the input data, edge weights for the regulatory network are reported for all *m *×* n* TF–gene edges.

The distribution of these edge weights has approximate mean 0 and standard deviation 1. The edges are therefore best interpreted in a relative sense. Strongly positive values are indicative of relatively greater evidence of a regulatory TF–gene association and smaller or negative values can be interpreted as lacking evidence of regulatory interaction. Consequently, users often want to see only a high edge weight subset of the complete network in order to focus on the most strongly supported regulatory interactions. This filtering is performed using the topedges function. A network containing the top 1000 edge scores as binary edges can be obtained using the command

topNet <– topedges(pandaResult, 1000)

The network can be further simplified to a TF set of interest by using the subnetwork method,

trFactors <– c(“TLX1”,”VDR”,”RXRA”,”PPARG”)

topSubnet <– subnetwork(topNet, trFactors)

to limit the output to a subset of TFs and the genes that they are found to regulate.

## Funding

This work was supported by the US National Institutes of Health, including grants from the National Heart, Lung and Blood Institute [5P01HL105339, 5R01HL111759, 5P01HL114501, K25HL133599]; the National Cancer Institute [5P50CA127003, 1R35CA197449, 1U01CA190234, 5P30CA006516]; and the National Institute of Allergy and Infectious Disease [5R01AI099204].

*Conflict of Interest*: none declared.
